# Multiple myeloma and physical activity: a scoping review

**DOI:** 10.1136/bmjopen-2015-009576

**Published:** 2015-11-27

**Authors:** Lee Smith, Orla McCourt, Malgorzata Henrich, Bruce Paton, Kwee Yong, Jane Wardle, Abigail Fisher

**Affiliations:** 1 Department of Epidemiology and Public Health, Health Behaviour Research Centre, University College London, London, UK; 2 Institute of Sport Exercise and Health, University College London, London, UK; 3 Research Department of Haematology, Cancer Institute, University College London, London, UK

**Keywords:** ONCOLOGY

## Abstract

**Objectives:**

Multiple myeloma is the second most common haematological cancer. A growing body of literature is emerging that investigates the role physical activity plays in all stages of multiple myeloma (prevention and survivorship) and to date no attempt has been made to collate and understand this literature. Therefore, this scoping review aims to (1) outline what is already known about physical activity in all stages of multiple myeloma (2) map the literature on physical activity and multiple myeloma and (3) identify future directions for research.

**Design:**

Scoping Review.

**Data Sources:**

Searches were carried out in May 2015. Searchers were conducted in PubMed, Web of Science, SPORTdiscus and MEDLINE.

**Eligibility criteria for selecting studies:**

To be included studies had to report original data, investigate physical activity per se or physical activity correlates and multiple myeloma or smouldering multiple myeloma.

**Results:**

A total of 19 papers received full screening, 5 of these papers were excluded. This review identified three journal articles relating to the role of physical activity in the prevention of multiple myeloma, nine papers were identified in the treatment of multiple myeloma and two on smouldering multiple myeloma.

**Conclusions:**

The search identified that the literature surrounding multiple myeloma and physical activity is very limited. We encourage those designing new cohort studies to allow for future assessment of associations between physical activity and onset of multiple myeloma and smouldering multiple myeloma, as well as the potential role that physical activity plays in the progression from smouldering multiple myeloma to multiple myeloma. Second, we encourage the design and investigation of gender and treatment-specific physical activity interventions in patients with multiple myeloma. Finally, we highlight the need for more randomised controlled trials to evaluate the impact of different types, frequencies and intensities of physical activity on various health parameters in multiple myeloma survivors.

**Strengths and limitations of this study**

- This is the first attempt to collate literature surrounding physical activity and multiple myeloma.
- There has been little research on physical activity for multiple myeloma and even less for smouldering multiple myeloma.
- This review has identified several avenues for new research on multiple myeloma and physical activity.
- This review is limited by a shortage of research.

## Introduction

### Multiple myeloma

Multiple myeloma (MM) is the second most common haematological cancer, resulting from the accumulation of malignant plasma cells in the bone marrow.^1^ MM is responsible for approximately 1% of all cancer cases globally and in the UK, and incidence rates have increased in the UK since the mid-1970s (http://www.cancerresearchuk.org). There is no known cure for MM, but current treatment modalities can control the disease for prolonged periods. MM is generally a disease of older adults, and approximately two-thirds of patients diagnosed are aged 65 years or more.^1^ Survival is variable, but about a third of adult patients with MM diagnosed in 2010–2011 in England and Wales are predicted to survive for 10 or more years (http://www.cancerresearchuk.org). Recent analyses have shown promising evidence of improved outcomes, likely owing to increasing use of novel treatment agents used during initial treatment such as thalidomide and bortezomib.^2^ MM is associated with a number of complications which include haematological complications (anaemia, bone marrow failure, bleeding disorders), bone complications (pathological fractures, lytic bone lesions, hypercalcaemia related to excessive bone turnover), renal insufficiency, compromised immune function and neurological complications (eg, spinal cord and nerve root compression and cranial nerve compression, often related to base of skull lesions).^3^

Almost all patients with MM will have had the precursor smouldering multiple myeloma (SMM); SMM is characterised by the presence of MM cells in the bone marrow and M-proteins in the blood, but without evidence of organ damage. Individuals with SMM have a 10–20% risk of developing MM each year.^4^ Recent recommendations by the International Myeloma Working Group (IMWG) identify a group of patients with SMM who are at a high risk of progression to MM, for whom systemic therapy is recommended.

Improved treatments for MM means patients with the disease are living longer, but with the cumulative burden of bone disease, compromised immunity and treatment-related toxicities, that prevent the return to good quality life. MM survivors often suffer from the sequelae of bone destruction: persistent deformities, chronic pain, reduced mobility and physical functioning. It could be hypothesised that participation in physical activity may be a beneficial strategy to improve reduced mobility and physical functioning. Hence there is an increasing need for non-pharmacological strategies for the management of this patient group.^2^

### Physical activity and the prevention of cancer

There is evidence from over 100 epidemiological studies, that routine physical activity is associated with the reduction in the incidence of some cancers (eg, breast and lung; see reviews by^5^ ^6^), there also appears to be a dose–response relationship,^7^ that is, the higher the intensity of routine physical activity the greater the protective effect against cancer. However, the benefits of physical activity may vary between cancer type, for example, a recent meta-analysis on physical activity in relation to risk of haematological cancer identified 23 papers and showed non-significant associations. When comparing high versus low physical activity levels, the RR for non-Hodgkin's lymphoma was 0.91 (95% CI 0.82 to 1.00), for Hodgkin's lymphoma it was 0.86 (95% CI 0.58 to 1.26), for leukaemia it was 0.97 (95% CI 0.84 to 1.13) and for MM it was 0.86 (95% CI 0.68 to 1.09).^8^

### Physical activity and cancer survivors

Focus has also turned to physical activity for cancer survivors. Physical activity has been shown to be safe and feasible during cancer treatment and can improve physical functioning and quality of life, as well as reduce fatigue.^9^ A recent meta-analysis of high-quality controlled trials^10^ identified the importance of regular participation in physical activity for the health of cancer survivors. The analyses showed that physical activity has been found to improve aerobic fitness, muscular strength, functional quality of life and self-esteem, and reduce anxiety. However, the majority of studies identified investigated solid tumours and very few investigated haematological cancers. A number of reviews have also been carried out on the role of physical activity in the survival of a number of specific cancers, including breast, lung and prostate.^11–13^ A growing body of literature is emerging that investigates the role physical activity plays in all stages of MM (prevention and survivorship) and to date no attempt has been made to collate and understand this literature.

### Aim

Scoping reviews are relatively novel, compared to other review types (eg, systematic) and are growing in popularity. Scoping reviews allow the researcher to (1) clarify working definitions and conceptual boundaries of a topic area, (2) outline what is already known and identify gaps in existing research, and (3) map existing literature of existing evidence.^14^ This scoping review aims to (1) outline what is already known about the role of physical activity in all stages of MM (2) map the literature on physical activity and MM and (3) identify future directions for research.

## Methods and analysis

### General framework for scoping review and initial search criteria

The main stages for a scoping review stated in Levac *et al*^15^ were followed (1) Identify the research question, (2) Identify relevant studies, (3) Study selection, (4) Charting the data, (5) Collating, summarising and reporting the results. The initial question was “what is known about the role of physical activity in all stages of MM (prevention and survivorship).” Studies including the MM precursor SMM were included since individuals have a 10–20% chance of developing MM each year.

Search criteria were then established; in order to scope and include the majority of literature the exclusion criteria were minimal and included (1) any study investigating physical functioning and not physical activity per se (2) non-human studies (3) studies not published in English, (4) and studies investigating only the benign condition Monoclonal Gammopathy of Undetermined Significance (MGUS) (since there is only a 1% chance a year of developing MM). To be included studies had to report original data, investigate physical activity per se or physical activity correlates and MM or SMM, work published in peer-reviewed academic journals, PhD dissertations, research reports and full conference papers were considered. Searchers were conducted in PubMed, Web of Science, SPORTdiscus and MEDLINE. MEDLINE and PubMed were both searched separately as a type of sensitivity analyses for the search terms, since PubMed is a search engine for MEDLINE; all papers identified in PubMed were identified in MEDLINE and vice versa. Search terms entered into each database are presented in table 1. Titles of identified papers were screened by one reviewer (LS) and those with titles not deemed relevant were removed. Remaining papers were then screened by two reviewers (LS and OM) against the inclusion/exclusion criteria, no disagreements occurred between reviewers on which papers to include. Reference lists of remaining papers after review were then searched to identify papers missed in electronic searches, and reference lists of those papers, and so on until no further papers could be identified.

**Table 1:** Search terms

PubMed	"multiple myeloma"[MeSH Terms] OR (“multiple"[All Fields] AND “myeloma"[All Fields]) OR “multiple myeloma"[All Fields]) AND (“motor activity"[MeSH Terms] OR (“motor"[All Fields] AND “activity"[All Fields]) OR “motor activity"[All Fields]
Web of Science	Myeloma and physical activity or exercise or walking
MEDLINE	Multiple myeloma and physical activity or exercise or walking
SportsDiscuss	Multiple myeloma and physical activity or exercise or walking

## Results

**Figure 1:**
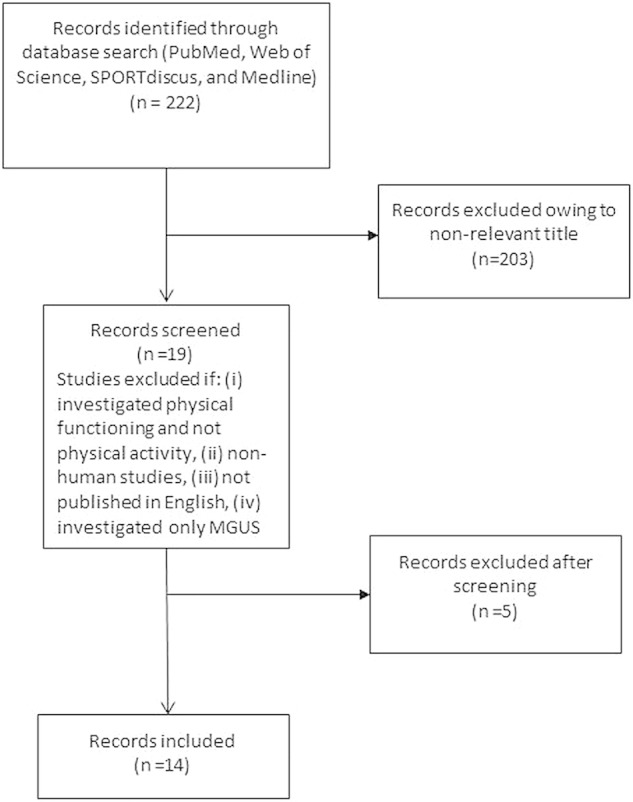
Flow chart of citations. MGUS, Monoclonal Gammopathy of Undetermined Significance.

Searches were carried out in May 2015. First the search engine PubMed was searched and 35 papers were identified, of which 13 were entered into full-text review. Next, Web of Science was searched and 134 papers were identified, of which an additional four, previously unidentified papers, were entered into full-text review. We then searched SPORTdiscuss and MEDLINE combined, 53 papers were identified and two additional papers (identified by SPORTdiscuss) were incorporated. A total of 19 papers were reviewed, 5 of these papers were excluded, 1 paper examined only physical functioning and not physical activity, another paper did not investigate MM or SMM per se, 1 paper did not distinguish MM from other haematological cancers, another was a letter and one was the reporting of a protocol. Finally, we searched reference lists of papers and no further studies were included. A total of 14 papers were included in this review. See figure 1 for flow of citations.

The following information was extracted from each paper: Title, author, year of publication, study design, study aim and key findings (tables 2–4). If the information was not available from the abstract the appropriate information was extracted from the full text. Findings from scoping the literature are presented as stage of cancer journey (prevention and survivorship). Results for MM and SMM are reported separately.

**Table 2:** Description of studies on physical activity and the prevention of multiple myeloma

Author	Year of publication	Study design	Sample	Study aim	Length of follow-up	Covariate adjustment	Key findings
Khan^17^	2006	Prospective cohort	109 698 Japanese adults aged 40–79 years	Assess the association of MM with physical activity in a Japanese cohort	15 years	Age and sex	For both sexes, walking ≤30 min/day significantly increased age and sex adjusted MM risk (HR=2.0; 95% CI 1.2 to 3.4)
Hofmann^16^	2013	Prospective cohort	305 618 US adults aged 50–71 years	To investigate the risk of MM in relation to physical activity at different ages in the National Institutes of Health-AARP Diet and Health Study	11 years	Age, sex, race and BMI	Risk of MM was not associated with physical activity level at any age
Birmann^18^	2007	Prospective Cohort	121 700 US registered nurses aged 30–55 years and 51 529 US male health professionals aged 40–75 years	To examine the role of energy balance in MM aetiology in two large prospective cohorts with biennially updated exposure data	22 years and 16 years	Age and BMI	Physical activity was not significantly related to MM risk. Although an inverse association was suggested in women

BMI, body mass index.

**Table 3a:** Description of qualitative and cross-section studies on physical activity and multiple myeloma survivorship

Author	Year of publication	Study design	Study aim	Key findings
Craike^21^	2013	Qualitative semistructure telephone interview	To gain an in-depth understanding of the physical activity experiences and perceived benefits and barriers to physical activity for patients with MM	Barriers to physical activity predominately related to the symptoms of MM and side effects of therapy, including pain, fatigue and fear of infection Low self-motivation was also a barrier Women participated in a more diverse range of physical activities than men and there were gender differences in preferred type of physical activity Women were more likely to report psychological and social benefits; whereas men reported physical activity as a way to keep busy and self-motivation was a barrier
Coon^19^	2004	Qualitative—face to face interviews of intervention participants in RCT	The aim of this study was to understand how participants decide when to start, interrupt, stop or resume an exercise programme or adjust the intensity. And to understand what helped or hindered the participants’ ability to do exercises	Intrinsic factors that facilitated exercise adherence included a belief that exercise would be beneficial, a personal moral/ethical philosophy with regard to honouring a commitment and/or taking responsibility for one's health, and personal strategies such as keeping a routine and setting goals Extrinsic facilitators included having a good support system and receiving prophylactic epoetin alfa
Coon^20^	2004	Qualitative interviews—naturalistic (constructionists) of participants taking part in an exercise programme	To ascertain how patients with MM appraised the experience of participating in a home-based exercise intervention as part of a RCT of prophylactic epoetin alfa with or without exercise	Patients with MM can safely carry out a home-based exercise programme aimed at reducing cancer-related fatigue Commitment to keeping their promise to exercise helped participants to continue their exercise programme during times when they were not feeling well Encouragement from family and healthcare professionals facilitates adherence to an exercise programme Many participants avoided taking time off from exercise because they had experienced dramatic declines in their strength and stamina after interruptions to their exercise programme However, most patients needed to reduce the exercise intensity or take time off from exercise immediately following chemotherapy
Craike^26^	2013	Cross-sectional survey, including retrospective recall	To examine, for people treated for MM, (1) differences between prediagnosis and postdiagnosis levels of physical activity, (2) perceived barriers and likelihood of attending a physical activity programme and (3) factors that influence whether or not respondents are meeting physical activity guidelines	Significantly more people were meeting physical activity guidelines prediagnosis (38.9%) than currently (20.1%) The strongest perceived barrier was fatigue (37.8%), followed by injuries (34.2%), pain (28.1%), other health conditions (21.1%) and age-related decline in physical ability (18.5%). Lack of knowledge about physical activities that are safe (19.7%), lack of confidence in physical ability (17.1%) and fear or injury (16.2%) were also reported Perceived barriers relating to interpersonal factors were least likely to be reported as barriers to participation, including nausea (7.9%), cost of exercise (9.2%), no one to exercise with (10.1%), and lack of time (10.1%) When asked how likely they would attend an exercise programme designed for people with MM, 41.1% reported they would be very likely or extremely likely to attend and 41.1% said they would not at all likely or slightly likely to attend Only prediagnosis levels of physical activity significantly predicted current levels of physical activity. Overall, people who participated in sufficient levels of physical activity prior to their diagnosis were 4.79 times more likely to meet physical activity guidelines compared to people who did not meet guidelines prior to diagnosis
Jones^27^	2004	Cross-sectional survey, including retrospective recall	To examine the association between exercise and quality of life in multiple myeloma cancer survivors	Descriptive analyses indicated that 6.8% and 20.4% of survivors met national exercise guidelines during active and off-treatment periods, respectively Exercise during active treatment and off-treatment were positively associated with overall quality of life

RCT, randomised controlled trial.

**Table 3b:** Description of experimental studies on physical activity and multiple myeloma survivorship

Author	Year of publication	Study design	Study aim	Key findings
Coleman^23^	2012	RCT with repeated measures 15-week intervention period Intervention consisted of stretching, strength and resistance, and aerobic exercise (frequency of exercise sessions and duration is not reported)	To compare usual care with a home-based individualised exercise programme (HBIEP) in patients receiving intensive treatment for MM and epoetin alfa therapy	No statistically significant differences existed among the experimental and control groups for fatigue, sleep or performance (aerobic capacity) (p>0.05) Exercise combined with epoetin alfa helped alleviate anaemia
Coleman^22^	2008	RCT 15-week intervention period Intervention consisted of stretching, strength and resistance, and aerobic exercise (frequency of exercise session and duration is not reported)	To determine the effect of epoetin therapy (short term vs long term) with and without a home-based individualised exercise programme that incorporated aerobic and strength resistance training for patients being treated with high-dose chemotherapy and autologous peripheral-blood stem cell transplantation for MM	Recovery and treatment response were not significantly different between groups after transplantation The exercise group had significantly fewer red blood cell transfusions and fewer attempts at stem cell collection (p<0.025) Serious adverse events were similar in each group (p>0.05)
Coleman^24^	2003	RCT Intervention period, session frequency and duration is not reported Intervention consisted of stretching, strength and resistance, and aerobic exercise	To test the feasibility of home-based exercise therapy for patients receiving high-dose chemotherapy and autologous peripheral blood stem cell transplantation as treatment for MM	Individuals assigned to exercise gained an average lean body weight of 0.40 kg per month The non-exercise group experienced and average decline in lean body weight of −0.44 kg/month (p<0.01) The study suggests that an exercise programme for patients receiving aggressive treatment for MM is feasible and may be effective in decreasing fatigue and mood disturbance while improving sleep
Groeneveldt^25^	2013	Single arm pilot study 6-month intervention period Intervention consisted of taking part in stretching, strength and resistance, and aerobic exercise three times a week	To assess the feasibility (accrual rate, acceptability and adherence) and safety of an exercise programme for MM survivors	The exercise programme was feasible and safe for patients with MM and there was high attendance and acceptability Benefits were evident in patients reported outcomes of QOL, fatigue and measured muscle strength (all p<0.01)

RCT, randomised controlled trial; QOL, quality of life.

**Table 4:** Description of studies on physical activity and smouldering multiple myeloma

Author	Year of publication	Study design	Study aim	Key findings
Boullosa^28^	2010	Case Report	To document heart rate variability (HRV) during a 6-week period in a female SMM patient that was currently performing a high-intensity training programme	The major finding was the reported similar HRV for a highly physically active young SMM patient to that of age-matched controls
Boullosa^29^	2013	Case Report	To evaluate the influence of a supervised training programme on the changes in serum monoclonal protein level (ie, IgG), percentage of bone marrow plasma cells (BMPCs), fitness performance and cardiac autonomic control (ie, HR variability (HRV) and HR complexity (HRC) in a female diagnosed with SMM	Exercise performance in all fitness components was improved while IgG levels and BMPCs decreased from 20% to 10%, respectively Time and frequency domain HRV parameters exhibited significant increases (18%–29%) with HRC remaining unchanged The current case report results indicated that a multimodal training programme designed for the development of various physical capacities improved exercise performance, haematological function and cardiac autonomic control that may improve long-term prognosis for SMM

### Prevention

This review identified three journal articles relating to the role of physical activity in the prevention of MM (table 2). All studies were of a prospective cohort design with 11,^16^ 15 17 and 22 years 18 follow-up, these studies produced mixed results. Two studies^16^ ^18^ found no associations between baseline physical activity and incident MM. One study found that for men and women walking less than 30 min a day at baseline, compared to walking greater than 30 min a day, was associated with increased age and sex-adjusted MM risk (HR=2.0; 95% CI 1.2 to 3.4). 17

### Survivorship

Nine papers were identified that addressed physical activity during, or after, treatment for MM, of which three used a qualitative design,^19–21^ three used a randomised controlled trial (RCT) 22–24 one was a single arm pilot study,^25^ and two were cross-sectional^26^ ^27^ (tables 3 and 4).

The qualitative studies aimed to identify barriers and facilitators to physical activity in patients with MM. Barriers to physical activity predominately related to the symptoms of MM and side effects of therapy, including pain, fatigue and fear of infection. Low self-motivation was also a barrier. Women participated in a more diverse range of physical activities than men and there were gender differences in preferred type of physical activity. Women were more likely to report psychological and social benefits; whereas men reported physical activity as a way to keep busy and self-motivation was a barrier. Patients treated with an autologous stem cell transplant more often reported affective benefits of participation in physical activity and fatigue as a barrier. Patients treated with other (non-transplant related) therapies (eg, chemotherapy, radiotherapy) were more likely to report pain as a barrier. Commitment to keeping their promise to exercise helped patients to continue their exercise programme during times when they were not feeling well. Encouragement from family and healthcare professionals was reported to facilitate adherence to an exercise programme. Patients reported needing to reduce the exercise intensity or take time off from exercise immediately following chemotherapy.

One cross-sectional study^26^ aimed to examine the differences between prediagnosis and postdiagnosis levels of physical activity using retrospective recall, perceived barriers and likelihood of attending a physical activity programme, and factors that influence whether or not respondents are meeting physical activity guidelines. The study found that significantly more people reported meeting the physical activity guideline of 150 min of moderate-to-vigorous activity per week prediagnosis than post. Those who participated in sufficient levels of physical activity prior to diagnosis were approximately five times more likely to meet physical activity guidelines postdiagnosis, compared to people who did not meet guidelines prior to diagnosis. Reported perceived barriers to physical activity participation were fatigue, injuries, pain, other health conditions, age-related decline in physical ability, lack of knowledge on physical activities that are safe, cost of exercise, nausea, no one to exercise with and lack of time. The other cross-sectional study examined the association between physical activity and quality of life in MM survivors. It was found that 6.8% and 20.4% of survivors met physical activity guidelines during active and off treatment periods, respectively. Participation in physical activity was found to be positively associated with overall quality of life.^27^

The single arm pilot study explored the feasibility and patient outcomes of a tailored, physiotherapist-led exercise programme, consisting of stretching, strength and resistance, and aerobic exercise, in treated MM survivors.^25^ This exercise programme was found to be feasible and safe with no adverse reactions to exercise reported. Uptake to the exercise programme and attendance rates were high indicating willingness to engage and strong adherence to the physical activity intervention. Benefits were demonstrated in patient reported quality of life and improvements in upper and lower limb strength.

One RCT aimed to determine the effect of epoetin therapy with and without a home-based individualised exercise programme that incorporated stretching, aerobic and strength resistance training for patients being treated with high-dose chemotherapy and autologous peripheral blood stem cell transplantation for MM.^22^ The study found that recovery and treatment response were not significantly different between groups after transplantation. However, the exercise group had significantly fewer red blood cell transfusions and fewer attempts at stem cell collection. Another RCT 23 aimed to compare usual care with a home-based individualised exercise programme, consisting of stretching, strength and resistance, and aerobic exercise, in patients receiving intensive treatment for MM and epoetin alfa therapy. No differences were found between the experimental and control groups for fatigue, sleep or performance (aerobic capacity). However, exercise combined with epoetin alfa helped alleviate anaemia. One RCT^24^ included participants receiving high-dose chemotherapy and autologous peripheral blood stem cell transplantation and tested the feasibility of home-based exercise therapy (consisting of similar exercises to those previously mentioned). The study found that those assigned to the exercise group experienced a significant increase in lean body weight compared to the non-exercise group. Moreover, the study suggests that an exercise programme for patients receiving aggressive treatment for MM is feasible and may be effective in decreasing fatigue and mood disturbance while improving sleep.

### Physical activity and SMM

The scoping review identified just two papers on SMM and physical activity, both of these were case reports on the same female patient with SMM. One case study aimed to document heart rate variability during a 6-week period in a female patient with SMM who was currently performing a high-intensity training programme.^28^ The patient with SMM recorded similar heart rate variability to that of six age-matched controls. The other case study^29^ aimed to evaluate the influence of a training programme on changes in serum monoclonal protein level, percentage of bone marrow plasma cells, fitness performance and cardiac autonomic control. The case study reports that exercise performance in all fitness components improved while serum monoclonal protein levels and percentage of bone marrow plasma cells decreased. Moreover, time and frequency domain heart rate variability parameters exhibited significant increases with heart rate complexity remaining the same.

## Discusion

This scoping review has highlighted that to date there has been little research on physical activity for MM and even less for SMM. The literature that does exist on the role of physical activity in the prevention of MM is inconclusive and much more epidemiological research is needed in this area using larger samples and prospective cohort designs. We encourage those in the process of designing new cohort studies to allow for future investigation of such longitudinal associations.

This review has identified numerous suggested social, sociodemographic and biomedical barriers and facilitators to physical activity in patients with MM. Identified barriers and facilitators suggest that gender and treatment-specific physical activity interventions may yield the best results. We recommend that future research should test this hypothesis. Furthermore, those who have low levels of physical activity prediagnosis may benefit the most from physical activity interventions. More research is needed to investigate the generalisability of the identified barriers and facilitators to other populations of patients with MM, and longitudinal studies to address associations are encouraged.

There is limited evidence using RCTs to evaluate the impact of physical activity on health parameters in patients with MM during and after treatment, although it appears to be safe and accepted by patients. Existing evidence from RCTs suggests physical activity may aid in various MM treatments and alleviate some comorbidities. When reviewing the literature one protocol of an ongoing study was identified that aims to determine the effectiveness of an individualised high-intensity strength and interval training programme with respect to physiological and psychological status in patients with MM, who have recently undergone high-dose chemotherapy followed by autologous stem cell transplantation, using a RCT.^30^ Future research should include RCTs to evaluate the impact of different types, frequencies and intensities of physical activity (eg, aerobic or anaerobic) on various health parameters in patients with MM (eg, fatigue, quality of life, sarcopenic obesity, bone health and anaemia). Of specific interest may be the effect of physical activity on the toxicities experienced by patients receiving anti-MM therapies, as well as the effect of particular drugs or treatment regimens on levels of physical activity. Such questions will become increasingly important with the improved survival of patients with MM, and the trend towards extended therapy. However, it should be noted that one important concern with MM in regard to physical activity is the fracture risk related to bone lesions. A fracture risk assessment should be carried out on a patient with MM prior to being given permission to be physically active. This has been performed in the past using radiographs in combination with Mirel's score. For example, in a recent study by Groeneveldt *et al*, radiographs were assessed for fracture risk by a multidisciplinary team of musculoskeletal radiologist, clinical oncologist, myeloma specialists, physiotherapist and clinical nurse specialists. Patients considered to be at risk of fractures, for example, with large lytic lesions of the long bones or extensive lytic disease in the pelvis, underwent cross-sectional imaging with CT or MRI and were referred for surgery and/or radiotherapy. Those not at risk were recommended for exercise, while some of those at risk underwent surgical fixation before embarking on the exercise programme.^25^

Only two studies were identified in the area of SMM and physical activity. However, inferences should not be drawn from two case studies on a single patient with SMM. This is an understudied research area and one that requires epidemiological investigation.

This is the first review of literature on physical activity and MM and SMM. The variety of search databases utilised, as well as extensive reference searches, reduced the risk of bias. However, there is a potential influence of publication bias, with negative and null findings remaining in the ‘file drawer.’ This review is limited by a shortage of research and it should be noted that further data may exist. This review did not include studies in which MM was pooled with other haematological cancers.^31^ ^32^ Moreover, the use of additional search terms may have identified other relevant papers.

## Conclusion

The literature surrounding MM and SMM and physical activity is limited. Much more research is needed in all areas of MM and SMM and physical activity to understand the true benefit of physical activity for this population. We encourage those in the process of designing new cohort studies to allow for future assessment of associations between physical activity and onset MM and SMM, as well as the potential role physical activity plays in the transition from SMM to MM. Second, we encourage the design and investigation of gender and treatment-specific physical activity interventions in patients with MM. Finally, we highlight the need for more RCTs to evaluate the impact of different types, frequencies and intensities of physical activity on various health parameters in patients with MM.
